# Alarmins in cutaneous malignant melanoma: An updated overview of emerging evidence on their pathogenetic, diagnostic, prognostic, and therapeutic role

**DOI:** 10.1111/1346-8138.17278

**Published:** 2024-05-22

**Authors:** Vincenzo Papa, Federica Li Pomi, Francesco Borgia, Mario Vaccaro, Giovanni Pioggia, Sebastiano Gangemi

**Affiliations:** ^1^ Department of Clinical and Experimental Medicine, School and Operative Unit of Allergy and Clinical Immunology University of Messina Messina Italy; ^2^ Department of Precision Medicine in Medical, Surgical and Critical Care (Me.Pre.C.C.) University of Palermo Palermo Italy; ^3^ Department of Clinical and Experimental Medicine, Section of Dermatology University of Messina Messina Italy; ^4^ Institute for Biomedical Research and Innovation (IRIB), National Research Council of Italy (CNR) Messina Italy

**Keywords:** alarmin, HMGB1, IL‐1, IL‐33, melanoma

## Abstract

Malignant cutaneous melanoma is the leading cause of death for skin cancer to date, with globally increasing incidence rates. In this epidemiological scenario, international scientific research is exerting efforts to identify new clinical strategies aimed at the prognostic amelioration of the disease. Very promising and groundbreaking in this context is the scientific interest related to alarmins and their pioneering utility in the setting of the pathogenetic understanding, diagnosis, prognosis, and therapy for malignant cutaneous melanoma. However, the scientific investigations on this matter should not overlook their still well‐presented dual and contradictory role. The aim of our critical analysis is to provide an up‐to‐date overview of the emerging evidence concerning the dichotomous role of alarmins in the aforementioned clinical settings. Our literature revision was based on the extensive body of both preclinical and clinical findings published on the PubMed database over the past 5 years. In addition to this, we offer a special focus on potentially revolutionary new therapeutic frontiers, which, on the strength of their earliest successes in other clinical areas, could inaugurate a new era of personalized and precision medicine in the field of dermato‐oncology.

## 1 INTRODUCTION

Cutaneous melanoma is a malignant neoplasm originating from the abnormal growth of melanocytes which, despite being relatively rare compared to other skin tumors, represents the main cause of mortality in skin cancer, with an increasing incidence worldwide. This situation highlights the critical need for a deeper understanding of the disease, its pathogenesis, development, and targeted therapeutic strategies.^1^ Recent research has highlighted the existence of a complex interaction between the immune system and the progression of melanoma, an observation further supported by the different responses to immunotherapy in each patient.^2^ From this starting point, research has focused on the role of alarmins and their ability to modulate the tumor microenvironment (TME), a complex ecosystem in which tumor cells interact with different cell types, including immune cells, fibroblasts, and endothelial and extracellular matrix system components. The term “damage‐associated molecular patterns” (DAMPs) has recently been used interchangeably with the term “alarmin.” However, some researchers consider alarmins to be a subset of endogenous DAMPs, produced specifically in response to cell death. Alarmins include various molecules including high mobility group box‐1 (HMGB1), S100 proteins, antimicrobial peptides (AMPs), heat shock proteins (HSPs), and members of the interleukin (IL)‐1. These molecules are endogenous proteins or peptides released by stressed, damaged, or dead cells, which act as danger signals, that in turn activate the immune system, thus stimulating neutrophils, monocytes, and macrophages, and subsequently releasing inflammatory mediators.^3^, ^4^ Alarmins appear to play a dual role in melanoma, acting as inflammation initiators and as facilitators of tumor immune evasion, thus leading to metastasis processes, while also playing an anti‐tumor role in some contexts. Emerging, albeit initial, evidence suggests that alarmins may serve as prognostic biomarkers and therapeutic targets in association with objectives already “targeted” by approved therapies in treating melanoma including immunotherapy. Despite research trying to elucidate the role of alarmins in melanoma progression, several key questions remain unanswered.

Therefore, through a synthesis of recent findings from preclinical and clinical studies, we seek to shed light on the complex interplay between alarmins and melanoma progression, focusing on potential therapeutic interventions. Ultimately, a deeper understanding of these processes may pave the way for the development of innovative immunotherapeutic approaches aimed at providing patient‐tailored therapies.

### 1.1 IL‐1

Autocrine secretion of IL‐1α in amoeboid melanoma cells plays a key role in enhancing the positive feedback crosstalk between rho‐associated protein kinase (ROCK)‐Myosin II and proinflammatory nuclear factor κB (NF‐κB), that supports tumor invasion activity and metastasis.^5^ What is more, in TME, the pro‐tumoral activity of IL‐1α is also extended to its key role in favoring immunotherapy resistance and suppression of anti‐tumor immunity via polymorphonuclear myeloid‐derived suppressor cells (PMN‐MDSCs).^6^ In such a TME, the increased accumulation and effective trafficking of PMN‐MDSCs is triggered and maintained by the IL‐1 signaling cascade itself through the downstream release of PMN‐MDSC‐recruiting chemokines.^7^ These findings are further explained by the recognized role of myeloid differentiation primary response 88 (MyD88)/IL‐1 receptor (IL1R) axis in maintaining the immunosuppressive function of tumor‐associated macrophages (TAMs).^8^ Contrary to many reports, melanoma cells are not the main source of IL‐1β due to the absence of inflammasome components.^9^ More recent findings emphasize the key role of nucleotide‐binding domain, leucine‐rich containing family, pyrin domain‐containing‐3 (NLRP3)/IL‐1β signaling in inducing the expansion of PMN‐MDSCs and subsequent suppression of anti‐tumor immunity in cutaneous melanoma.^10^ The just‐mentioned immunosuppressive mechanism in the tumor microenvironment is supported by an autoinflammatory loop regulated via the IL‐1β/IL‐6/signal transducer and activator of transcription 3 (STAT3) axis.^11^ Furthermore, moving among the 11 members of the large IL‐1 cytokine family, alongside the anti‐tumoral immunity suppressive role of IL‐38, also well investigated in melanoma, is the multilineage (both innate and adaptive) immunosuppressive role of IL‐1 through upregulation of Il‐37 in various lymphocyte subsets, notably in regulatory T (Treg) cells.^12^, ^13^ In TME, IL‐1β activating suppressor of mothers against decapentaplegic/DNA‐binding 1 protein inhibitor, the Smad/ID1 signaling pathway ensures maintenance of the stemness traits of melanoma cells, thus acting as a promoter of tumor relapse and metastasis. This is in addition to its recently investigated role promoting local invasion by increased expression of matrix metalloprotease (MMP)‐3 via p65/RelA in melanoma cells.^14^, ^15^ In the wake of the above, it must also be taken into account that an enhanced tumor aggressiveness is influenced by the presence of gene polymorphisms (specifically single‐nucleotide variations) of IL‐1β and IL‐1 type II receptor, an endogenous inhibitor of IL‐1β signaling.^16^ Exploring the reverse of the coin, alongside the already investigated role of IL‐1β in revitalizing the anti‐tumor CD8^+^ T cell response, pioneering is in melanoma the demonstrated anti‐tumor role of IL‐1β in inducing the differentiation of IL‐9‐producing CD4^+^ T‐helper (Th9) cells by exploiting an alternative signaling pathway. This would lead to the establishment of Th9 cells that were operationally less exhausted and with a more potent cytolytic transcriptional signature than the classic population with the ultimate result of more aggressive tumor‐specific killing activity.^17^, ^18^

### 1.2 HSPs

The heat shock response, and thus the downstream expression of HSPs in melanoma cells, is strongly influenced by changes in plasma membrane integrity, especially concerning cholesterol pools.^19^ In melanoma, the dual and contradictory role of many HSPs is well recognized, especially in their interaction with toll‐like receptors and in the regulation of immunological signaling pathways.^20^ HSP60, as a mitochondrial protein involved in energy metabolism, is overexpressed in the metabolic dysregulation framework of melanoma cells and associated with a poor clinical outcome.^21^ A recognized pro‐tumoral role of HSP60 calls into play its mitochondrial involvement in the CD147‐HSP60‐adenosine triphosphate (ATP)5B axis, resulting in activation of mitochondrial aerobic oxidation and increased ATP production, promoting melanoma cell invasiveness and migration.^22^ In melanoma, circulating heat shock protein 70 (HSP70) as the primary effector of NLRP3 triggering a distant TLR4‐dependent signaling cascade (NLRP3‐HSP70‐TLR4 axis), regulates granulopoiesis and recruitment and the accumulation of PMN‐MDSCs in distant organs, thus supporting the creation of a premetastatic niche.^23^ Furthermore, in the context of the well‐investigated anti‐apoptotic role in melanoma cells of HSP70,^24^ the 78 kDa cell surface glucose‐regulated protein (csGRP78), as a member of the HSP70 family, is involved in autoimmune‐mediated activation of signaling pathways promoting cancer cell survival.^25^ Concerning the presumed HSP90 role in melanoma, the scientific literature to date has been inconclusive.

There is evidence that overexpression of HSP90α correlates with prognostic worsening in malignant melanoma; also considered is the involvement of HSP90 clients in multiple drug resistance mechanisms.^26^, ^27^ In fact, HSP90α (also known as HSP86), in both structural (via tumor‐derived extracellular vesicles, [TEVs]) and soluble forms (via TLR4 signaling), also plays a key role in suppressing anti‐tumor immunity in melanoma by promoting the development of MDSCs.^28^, ^29^ In contrast, an important anti‐tumor role of HSP90 is demonstrated in human melanoma cells by interaction of this alarmin with the tumor‐suppressing Fhit protein and subsequent suppression of the oncogenic C‐Raf pathway.^30^ The dual and contradictory role of HSP90 is also demonstrated by the protective action of Hsp90/Cdc37 complex toward ubiquitination and proteasomal degradation of mixed lineage kinase 3, the loss of which promotes melanoma cell invasion via interaction between Hsp90/Cdc37 and BRAF and extracellular signal‐regulated kinases (ERK) hyperactivation.^31^ A key role of HSP90 in melanoma progression has recently been related to its angiogenesis‐promoting ability via activation of the IκB kinase (IKK)/IκB/NF‐κB/C‐X‐C Motif Chemokine Ligand 1 axis in cancer‐associated fibroblasts (CAFs) in the context of delivery of the HSP90/p‐IKKα/β complex by hypoxic TEVs.^32^ In melanoma, the role of HSP27 in promoting cancer cell migration and invasion through a mechanism of prion protein‐dependent F‐actin depolymerization is also recognized.^33^ On the other hand, in melanoma cells, evidence is provided for an anti‐tumor role of heat shock protein B8 (HSPB8) through arresting cell growth, cell migration, and reversion of the epithelial‐mesenchymal transition. Moreover, in melanoma cells with BRAF and NRAS mutations, HSPB8 exerts this role through the induction of autophagy, which counteracts cell growth.^34^ Research in this field continues to produce significant results, as evidenced by the recent finding of the anti‐tumor role from the interplay between HSPB8 and BAG Cochaperone 3 in blocking the oncogenic PI3K/AKT/mTOR pathway, thus deterring the survival, growth, and proliferation of cancer cells, thereby promoting their apoptosis and autophagy.^35^ In conclusion, investigation into the overexpression of HSP105/110 in cutaneous malignant melanoma found close associations with clinicopathological variables such as exposed site lesions, recurrent and metastatic lesions, nodular melanoma, and lentigo maligna melanoma.^36^

### 1.3 MMP‐2

In human melanoma, MMP2 appears dramatically overexpressed in metastatic melanoma cells compared to primary tumor cells.^37^ In this context, MMP2 would be useful as a valid prognostic biomarker,^38^ faithfully representing the various stages of malignant progression.^39^ However, overexpression of MMP2 is closely influenced by upregulation of N6‐adenosine‐methyltransferase.^40^ The production of this alarmin is also under the direct control of another alarmin, IL‐33, via the IL‐33/ST2/ERK1/2 signaling pathway.^41^ More and more emerging evidence proves the pro‐oncogenic, and specifically immunosuppressive role, of MMP2 in melanoma, since its overexpression is closely related to the increased infiltration of CAFs. More specifically, MMP2 exerts an immunosuppressive role both directly (by acting as an autoantigen, thus evading immunosurveillance as well as negatively modulating tumor resident antigen‐presenting cells [APCs] and T cells via deleterious TLR2‐ and TLR4‐signaling) and indirectly (through the immunosuppressive action of CAFs).^42^, ^43^ Moreover, in such a framework of generalized melanoma cell invasiveness, MMP2 does not contribute to the initial breakdown of brain endothelial barrier.^44^ Interestingly in melanoma, the pro‐invasive role of MMP2 is stimulated by adipocytes, fostering its hyperactivity. Therefore, this alarmin could be considered a valuable therapeutic target, especially in obese cancer patients.^45^ Noteworthy in this aspect of melanoma, the growing and promising strand of phytotherapeutic research focusing on the identification of natural compounds that, among the various pharmacological effects, have revealed remarkable inhibitory activity toward MMP2.^46^, ^47^, ^48^, ^49^, ^50^, ^51^ On the other hand, the controversial and dual role of MMP2 emerges from its association with improved overall survival rates in melanoma patients with a higher mutation burden due to its activity of freeing extracellular matrix (ECM) structural protein mutants adjacent to MMP2‐sensitive sites. The high‐affinity interaction with Human leukocyte antigens (HLA) class I molecules would promote anti‐tumor T cell immune response.^52^

### 1.4 S100

The role of S100 proteins in melanoma has recently been reviewed, suggesting their close interaction with receptor for advanced gycation end products (RAGE) signaling.^53^, ^54^ There is a wide differential expression of S100 family genes in melanoma, which correlate with the degree of tumor invasion, and for this reason play an important prognostic role.^55^ Moreover, for S100A8 and S100A9, overexpressed in mestastatic melanoma, in addition to their prognostic role, a predictive role in immunotherapy response has been evidenced.^56^ In the context of melanoma cell metastasization, alongside advances involving this alarmin in the development of new tools for the detection of circulating melanoma cells, also^57^ From the pathogenetic perspective, light has been shed on the molecular mechanisms which would explain the so‐called “seed and soil” theory. This latter could be attributed to the interaction between the S100A8/A9 complex and individual S100 soil sensor receptors (SSSRs) within the S100A8/A9‐SSSRs' axis. This latter, via TLR4 signaling, upregulates cellular metastatic events, including epithelial‐mesenchymal transition, cell motility and invasiveness, and the creation of an inflammatory immune suppressive environment in metastatic organs.^58^ Some evidence suggests a certain involvement of S100A9 in inducing acquired resistance to BRAF inhibitors.^59^ Even more upstream, the crucial early role of these alarmins was revealed in inducing pneumotropic metastasis of melanoma cells via their interaction with melanoma cell adhesion molecules in the framework of the S100A8/A9‐MCAM axis.^60^ Regarding S100A6, its expression in mestastatic melanoma correlates with the survival time of patients and the corresponding thickness of the primary tumor. On the other hand, the lack of S100A2 expression represents an early event in the development of melanoma.^61^ Interestingly, the key role of S100A11 in facilitating communication between melanoma cells with different metastatic colonization efficiency has just emerged. Precisely, polymetastatic cells transfer their polymetastatic competence to oligometastatic cells via the synergy between their secretory protein (S100A11‐Sec23a) and the exosomal (miR‐487a‐5p) pathways.^62^ As for S100B, it is a valuable serum biomarker in the follow‐up of stage III and IV melanoma patients,^63^ particularly in early detection of progressive disease as well as in the prediction response to BRAF inhibitors and anti‐programmed cell death protein 1 (PD‐1) therapy.^64^, ^65^, ^66^ Concurrently, on the therapeutic front, new evidence suggests the therapeutic efficacy of the synergistic blockade of S100B and RAF pathways in restoring the onco‐suppressive activity of p53.^67^ In this regard, more recent evidence confirms the therapeutic efficacy of S100B suppression in the recovery of intracellular free wild‐type‐p53 and subsequent restoration of the apoptotic signaling cascade in melanoma cells.^68^ The pro‐tumoral activity of S100B is also carried out in early‐stage melanoma through downregulation of IL‐6 signaling and subsequent suppression of anti‐tumoral immunity.^69^

### 1.5 AMPs

In the context of the broader investigation of both pro‐tumoral and antineoplastic roles of AMPs in skin cancers, alarmins are worthy of attention in melanoma. Specifically, the most recent observations on the subject do not yet allow us to define the precise pathogenetic role of human β‐defensins (hBD) in melanoma, although the hypo‐expression of hBD‐1 in melanoma patients compared with healthy controls and the reported antiproliferative effects of hBD‐2 on human melanoma cells in vitro suggest an anti‐tumor role of these two alarmins. Based on these functional assumptions, the development of new biotherapeutic strategies based on the use of viral vectors expressing β‐defensin 2 for melanoma is gaining ground.^70^ Interestingly, there have also been investigations into the cell‐killing potential of plant‐derived defensins in melanoma based on membranolytic rather than apoptotic cytotoxic mechanisms.^71^ Contrarily, a tumorigenic effect in melanoma is observed for cathelicidin LL‐37. The underlying molecular mechanisms call into play the upregulation of epidermal growth factor receptor (EGFR) and EGFR2, thus defining for this alarmin a role as a putative growth factor for melanoma cells, promoting their proliferation, migration, and invasion. Beside this mechanism, LL‐37 also promotes malignant melanoma progression via Y box‐binding protein and NF‐κB activation. The flip side of the coin would also see a non‐negligible antitumorigenic role of LL‐37 in supporting the optimal killing activity of natural killer (NK) cells. In a more comprehensive circumstances of interaction between AMPs and immune cells, emerging insights into a fine crosstalk between these peptides and TME cells (especially concerning TAMs) should not be overlooked. In this setting, synergistically with complement components, there is evidence in various types of cancer for a facilitatory role of defensins and cathelicidins. This would seem to be primarily through chemotactic capabilities, on the mobilization of immune cells from the bone marrow and their recruitment into the TME.^72^, ^73^, ^74^, ^75^, ^76^ The first and most obvious evidence of such crosstalk in melanoma microenvironment calls into play the upregulated tumor‐derived cathelicidin‐related antimicrobial peptide (CRAMP), the mouse analog of LL‐37 peptide in humans, by emphasizing its key role in reshaping tumor‐infiltrated FoxP3+ Th17 cells from effector Th17 cells into immunosuppressive Th17 cells via CD73 expression.^77^

Furthermore, later evidence of hypo‐expression of human ribonuclease 7 in melanoma would suggest its role as a tumor suppressor. Concerning S100A7/psoriasin, microarray data revealed underexpression of this peptide in cutaneous melanoma patients compared with healthy patients.^78^

### 1.6 HMGB1 and HMG nucleosome‐binding protein 1 (HMGN1)

The key pathogenetic role of HMGB1 in cutaneous melanoma has been revised based on the emerging body of work in recent years, emphasizing its involvement in promoting neoangiogenesis, pro‐tumor immunity (via accumulation of IL‐10‐producing M2 macrophages) as well as production of pro‐inflammatory cytokines IL‐23 and IL‐17 and suppression of cytotoxic T lymphocytes (CTLs) activity (via the RAGE/HMGB1 axis), thus enhancing tumor growth alongside the metastatic phenotype. Furthermore the potential of this alarmin as a prognostic marker and predictor of therapeutic response has been highlighted as well as its pioneering use as a target for the development of biological and miRNAs‐based drugs.^79^ Also supporting the above‐mentioned new therapeutic horizons, is the most recent evidence for the influence of HMGB1 suppression on the induction of neoantigen‐specific T cell immunity.^80^ Among the high‐mobility chromosomal proteins, worthy of mention as an alarmin in melanoma is HMGN1. In the wake of the previously emphasized role of HMGN1 in promoting anti‐tumor immunity with a preferential Th1‐polarizing ability, only in the past year have there been the first experimental results regarding the design of an HMGN1‐based vaccine as part of a combinational immunotherapy, proven effective in eradicating 60%–80% of three different melanin‐producing mouse melanomas.^81^, ^82^

### 1.7 IL‐33

Interleukin‐33 is a member of the IL‐1 family involved in several inflammatory and autoimmune conditions, normally localized at high levels within the nuclei of several cell types, including stromal fibroblasts, perivascular cells, tumor cells, endothelial cells, adipocytes, and epithelial cells.^83^, ^84^ In the presence of necrotic or damaged cells, IL‐33 is released into the extracellular space and binds to a heterodimer, composed of the orphan receptor ST2 and the accessory protein of the IL‐1 receptor.^85^ This interaction, found in immune cells and macrophages, serves as an alarm signal for the presence of tissue damage or infection.^85^ In melanoma, IL‐33 seems to act on macrophages promoting the transition from the M1 to M2 subtype, thus protecting melanoma cells from killing mediated by tumor‐infiltrating lymphocytes (TILs) and promoting metastatic processes through the production of high levels of MMP‐9.^86^ From this, targeting components of the IL‐33‐macrophage‐MMP‐9 axis could represent a potential therapeutic approach toward improving anti‐tumor immune therapy.^87^

Interleukin 33, following interaction with ST2 and acting as a proinflammatory cytokine, can also shape the TME and, consequently, activate lymphoid cells and CD8^+^ T cells.^88^ In contrast, IL‐33 was found to induce an anti‐tumor effect in vivo in a melanoma mouse model in which group 2 innate lymphoid cells 2 and CD8^+^ T cells infiltrated the tumor tissue. However, IL‐33 did not act directly on CD8^+^ T cells because they lack the ST2 receptor for IL‐33, but expressed OX40 ligand (OX40L) and OX40, respectively. In vivo, blocking the OX40L‐OX40 interaction inhibited the anti‐tumor effect of IL‐33. Specifically, the co‐culture of OX40‐expressing CD8^+^ T cells with IL‐33‐stimulated OX40L‐expressing ILC2, promoted cellular activation and proliferation of CD8^+^ T cells. Therefore, the IL‐33‐ILC2 axis promotes CD8^+^ T cell responses through OX40/OX40L interaction and exerts an anti‐tumor effect.^88^ In the future, this axis could represent another possible target for tailored therapies, to be administered alongside immunotherapy already on the market. Other studies in mouse models demonstrated that overexpression of IL‐33 could inhibit melanoma lung metastasis by activating CD8^+^ T cells and NK cells.^89^ Tumor‐induced IL‐33 expression appears to promote anti‐melanoma immune responses via interferon (IFN)‐γ‐producing CD8^+^ T cells and NK cells,^90^, ^91^, ^92^ while exogenous IL‐33 has been found to exert anti‐melanoma effects through the release of eosinophils and dendritic cells (DCs).^92^, ^93^, ^94^ Furthermore, systemic administration of recombinant IL‐33 can induce an anti‐tumor effect through a CD8^+^ T cell‐dependent mechanism. Notably, recombinant IL‐33 therapy by activating myeloid DCs in tumor‐bearing mice restored anti‐tumor T cell activity and increased antigen cross‐presentation within the TME. IL‐33 receptors ST2 and, above all, MyD88, which was required for the IL‐33‐mediated increase in the number of myeloid DCs, cooperate to induce the expression of co‐stimulatory molecules on myeloid DCs in response to recombinant IL‐33, thus revealing a novel IL‐33‐ST2‐MyD88‐STAT1 axis that restores the activation and maturation of myeloid DCs in cancer.^92^ However, conflicting results are emerging regarding the pro‐tumor or tumor suppressor effects of IL‐33 on melanoma. Some studies suggest that systemic administration of IL‐33 limits the growth of primary melanoma, while intranasal IL‐33 promotes lung metastasis of melanoma, through altering immune cell activity.^95^ Furthermore, the effects of IL‐33 appear to be context‐dependent, with its expression level showing variable correlations with immune cell components in the TME and prognostic value in primary versus metastatic samples.^96^ Elevated levels of IL‐33 in primary melanoma samples appeared to not correlate with patient prognosis, while metastatic samples correlated with prolonged overall survival and progression‐free survival.^96^ Moreover, IL‐33 has been implicated in the pathogenesis of melanoma by promoting vascular mimicry (VM). It increases the proliferation, migration, and invasion of melanoma cells as well as VM tube formation through ST2, possibly via the induction of MMP‐2/9 production through ERK1/2 phosphorylation.^41^

### 1.8 Thymic stromal lymphopoietin (TSLP)

Thymic stromal lymphopoietin, a cytokine belonging to the 4‐helix bundle cytokine family and distantly related to IL‐7, is expressed by epithelial cells and keratinocytes, while its release is typically NFkB‐dependent in response to various stimuli, including mechanical injury, infection, and inflammatory cytokines release.^97^ Initially associated with atopic and allergological conditions, recent research has shed light on TSLP's potential involvement in carcinogenesis.^98^ While it has been implicated in promoting Th2‐type inflammation and tumor progression in various cancers, including cutaneous melanoma, conflicting data also suggest potential anti‐tumor activity.^98^, ^99^, ^100^ In the context of malignant melanoma, a study by Yao et al. investigated the role of TSLP in tumorigenesis and immune evasion mechanisms. Using genetically engineered mouse models and tumor cell grafting, the authors explored the interplay between melanoma cells, keratinocytes, and immune cells. By inducing BRAF 600E expression and silencing PTEN, they generated primary melanomas with high penetrance, short latency, and lymph node metastasis. The study revealed that TSLP produced by keratinocytes in response to signals from melanoma cells promoted melanoma progression and metastasis through its actions on immune cells. Specifically, TSLP signaling through TSLPR‐expressing DCs appeared to induce GATA3+ Tregs, which exhibited potent suppressive effects on CD8^+^ T cell proliferation and IFN‐γ production, thus suggesting that TSLP plays a crucial role in shaping a pro‐tumoral immune microenvironment in malignant melanoma.^101^

### 1.9 IL‐25

**FIGURE 1:**
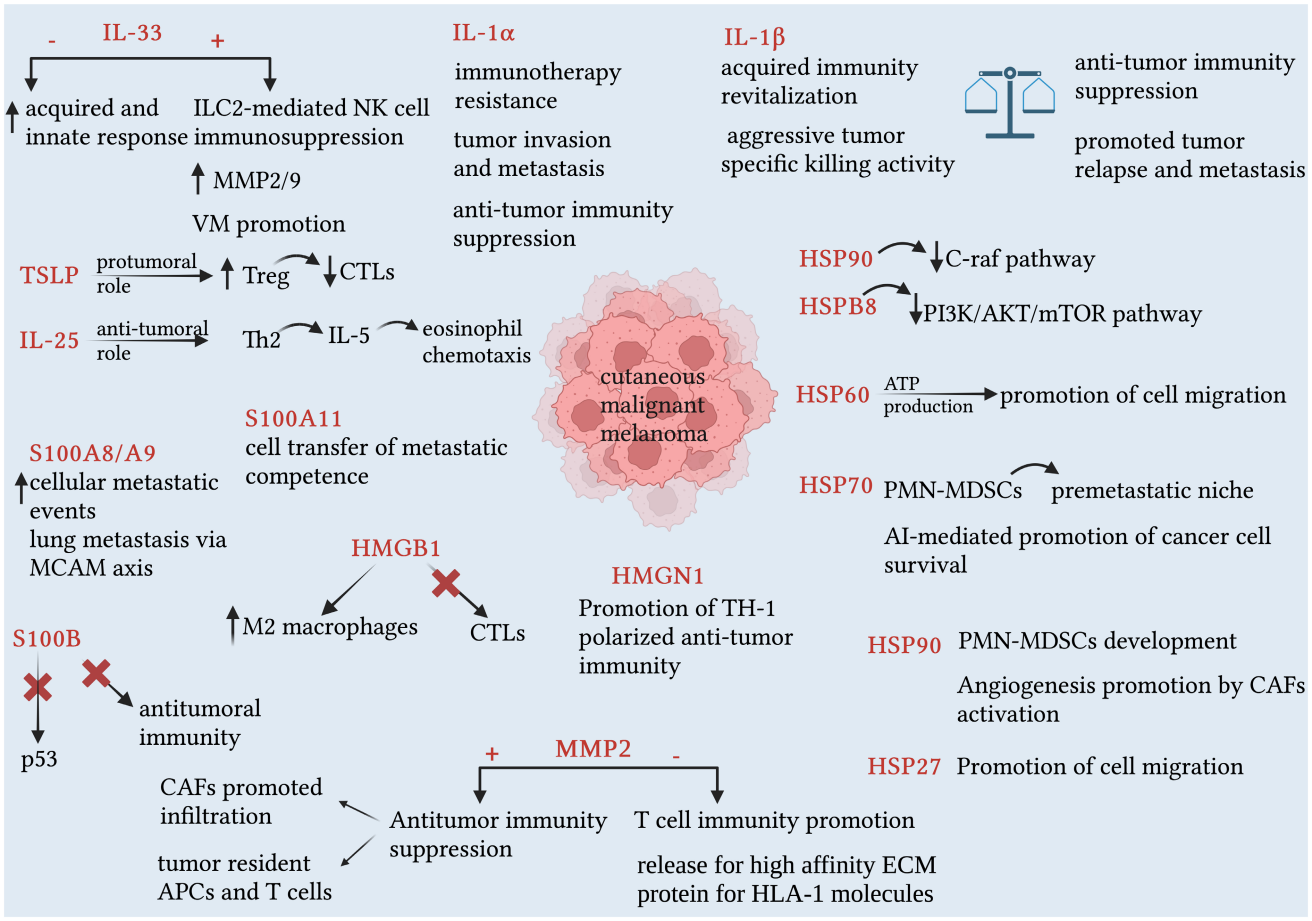
The main role of alarmins in cutaneous malignant melanoma. ATP, adenosine triphosphate; APCs, antigen‐presenting cells; CAFs, cancer‐associated fibroblasts; CTLs, cytotoxic T lymphocytes; ECM, extracellular matrix; HLA, human leukocyte antigens; HMGB1, high mobility group box‐1; HMGN1, high mobility group nucleosome‐binding protein 1; HSP, heat shock protein; IL, interleukin; ILC, innate lymphoid cell; MCAMs, melanoma cell adhesion molecules; MMP, matrix metalloprotease; PMN‐MDSCs, polymorphonuclear myeloid‐derived suppressor cells; TSLP, Thymic stromal lymphopoietin; VM, vascular mimicry.

Interleukin 25, belonging to the IL‐17 family of cytokines, acts through a receptor complex including IL‐17RA and IL‐17RB.^97^, ^102^ Upon binding of IL‐25, this receptor complex activates signaling pathways that culminate in the activation of NF‐κB, AP‐1, and other downstream proteins. To the best of our knowledge, only one study has been conducted on the role of IL‐25 in melanoma, in which overexpression of IL‐25 in mice resulted in a Th2‐type immune response, with expansion of eosinophils through increased IL‐5, IL‐4, and IL‐13 production. Injection of recombinant IL‐25 every other day produced significant anti‐tumor activity, while combining IL‐25 with chemotherapeutic or immunotherapeutic agents showed greater antitumor efficacy in human tumor xenograft models in mice compared to each agent administered alone. Tumor‐bearing mice treated with IL‐25 showed a significant increase in serum IL‐5 levels and an increase in the number of eosinophils in the peripheral blood compared to the control group. These data support the hypothesis of anti‐tumor activity of IL‐25 in vivo, paving the way for further investigations into its potential clinical use as an antitumor agent.^103^ Some preclinical studies have shown that Virulizin®, an anticancer and immunomodulatory agent prepared from bovine bile, can inhibit the growth of xenografted human tumor models, including melanoma, through the activation of NK cells and their infiltration into the TME. Further investigations indicated that Virulizin® stimulates the production of IL‐25 predominantly by B cells, resulting in elevated levels of eosinophils in the blood and infiltration of activated eosinophils into the TME.^104^ Although numerous studies have shed light on the role of IL‐25 in tumor immunity, further investigations are needed, particularly in human tumors, to elucidate its precise effects (Figure 1).

### 1.10 New emerging alarmins

In recent years, the potential tumorigenic role of transmembrane calreticulin has begun to be investigated in melanoma. In particular, a key immunomodulatory role has emerged from its promotion of macrophage polarization toward the M2 phenotype and subsequent release of transforming growth factor β, which promotes differentiation, followed infiltration of Foxp3+ Tregs into the TME, thus favoring tumor growth and dissemination.^105^ In this context, noteworthy in melanoma would be the emerging cytotoxic role of an endocannabinoid metabolite: 15‐deoxy, Δ12,14 prostamide J2 (15dPMJ2). The latter, acting as a sort of “super‐alarmin” would trigger, in an endoplasmic reticulum stress‐dependent manner, the exposure of damage‐associated molecular patterns (including cell surface CRT, ATP, HMGB1, HSP70 and HSP90) resulting in the recruitment and activation of APCs and activation of the anti‐tumor T cell immune response, thus eliciting an immunogenic cell death response.^106^

## 2 DISCUSSION

From our critical analysis focusing on the investigated functional role and related clinical importance of each alarmin in cutaneous malignant melanoma so far, a more conclusive view has emerged for some alarmins and less so for others. Concerning the IL‐1 family, the evidence of recent years about a pro‐tumoral role played by means of a positive crosstalk between proinflammatory (and more precisely, autoinflammatory) signaling, and mechanisms of anti‐tumor immunity suppression is clearly predominant. Given the promising use of anti‐IL‐1 drugs in other well‐known autoinflammatory disorders^107^ as well as the role of this alarmin in promoting immunotherapy resistance in melanoma, alongside its plausible clinical use as a predictive marker of immunotherapy response, a novel therapeutic choice in cutaneous melanoma could result from the use of anti‐IL‐1 drugs in combination with immunotherapy. However, from this perspective, the functional dichotomy of IL‐1β should be better investigated and resolved. Even more should be said, in view of this, about HSPs, specifically HSP70, that are also involved as an effector of NLRP3‐related pathways of inflammation as well as autoimmune pathways promoting tumor progression. However, the dualism should be further examined and clarified for HSP90 by better assessing its specific functional burden in suppressing oncogenic pathways and anti‐tumor immunity. More salient, are the latest findings about a role favoring cell invasion and migration in melanoma by HSP60, HSP27, and HSP105/110 as well as the promising anti‐melanoma role of HSPB8. As mentioned, functional dualism deserving of further investigation would also emerge for MMP2. Nonetheless, the available body of research in this regard still inclines toward its pro‐tumoral role, especially given the pioneering use of this alarmin as a therapeutic target in certain categories of cancer patients (such as the obese), along with the interest shown in it by the most recent phytotherapeutic research trends. On S100 proteins, the scientific literature to date has well emphasized their pro‐oncogenic in melanoma (namely, promoting metastatic colonization) and stressed the potential clinical utility of some of them, such as S100A8, S100A9, and S100B, as prognostic and predictive markers of therapeutic response in melanoma. Furthermore, the latest therapeutic achievements found by synergistic inhibition of S100B and RAF pathways in restoring the onco‐suppressive activity of p53 could be extended and replicated with a blockade of S100A9 as well the latter being plausibly involved in acquired resistance to BRAF inhibitors.

Similarly, pioneering in melanoma is the interest in the development of a new “alarmin‐based” vaccine, biotherapeutic, and phytotherapeutic strategies exploiting the immunoinductive, antiproliferative, and the cell‐killing potential that has emerged for HMGN1, hBD‐1, hBD‐2, and plant‐derived defensins. On the other hand, more elucidation on the functional contradiction in melanoma will have to concern the role of cathelicidin LL‐37. The functional dualism of the alarmins so far revisited goes hand in hand with their therapeutic dualism. Therefore, in view of this and in the wake of what has been proposed for anti‐IL‐1 drugs in the opening of this discussion, next to the new alarmin‐based approaches, a promising and feasible investigation scenario for the future might involve the use, also in cutaneous malignant melanoma and, more extensively, other intractable skin disorders,^108^ of anti‐alarmin drugs already employed and proven effective for other diseases. The numerous clinical trials underway are showing promising results regarding the use of anti‐epithelial‐derived alarmin drugs in various immune‐allergic disorders.^109^, ^110^ In this context, the anti‐inflammatory efficacy so far demonstrated in both T2‐high and T2‐low severe asthma and subsequent approval in several countries of the anti‐TSLP monoclonal antibody tezepelumab^111^ could pave the way for further investigations regarding the putative anti‐tumor immunity‐suppressive role of TSLP in melanoma, as well as the subsequent launch of clinical trials with the ultimate aim to achieve therapeutic approval of this biologic in the dermato‐oncological field. Similarly, early experimental results regarding an anti‐inflammatory role of HMGB1 inhibition in atopic dermatitis^112^ could prompt the development of an anti‐HMGB1 monoclonal antibody for that condition, thus further encouraging the already nascent research on HMGB1 use as a target in cutaneous melanoma (Table 1).

**TABLE 1:** Collection of the main articles included in the review and divided for each alarmin and possible role in cutaneous melanoma.

Author	Alarmin	Role in cutaneous melanoma
Georgouli et al.^5^ Singh et al.^6^	IL‐1α	**Pro‐tumoral** Promotion of tumor invasion by facilitating crosstalk between ROCK‐Myosin II and NF‐κB in amoeboid melanoma^5^ Promotion of immunotherapy resistance and suppression of antitumor immunity via PMN‐MDSCs^6^
Tengesdal et al.^10^ Lu et al.^14^ Nunomura et al.^15^ Van Den Eeckhout et al.^17^ Xue et al.^18^	IL‐1β	**Pro‐tumoral** Suppression of antitumor immunity via PMN‐MDSCs expansion^10^ Maintenance of melanoma cell stemness and promoted metastasis via Smad/ID1 signaling pathway^14^ Promoted local invasion with upregulation of MMP3 via p65/RelA^15^ **Anti‐tumoral** Revitalization of CTL‐mediated and Th9‐mediated antitumor immunity^17^, ^18^
Dowling et al.^12^ Osborne et al.^13^	IL‐37, IL‐38	**Pro‐tumoral** Anti‐tumoral immunity suppressive role^12^, ^13^
Lu et al.^22^	HSP60	**Pro‐tumoral** Promoted cell invasiveness by ATP production via CD147‐HSP60‐ATP5B axis^22^
Theivanthiran et al.^23^ Gonzalez‐Gronow et al.^25^	HSP70	**Pro‐tumoral** Recruitment of PMN‐MDSCs to distant organs via NLRP3‐HSP70‐TLR4 axis^23^ Autoimmune‐mediated promotion of cancer cell survival^25^
Arkhypov et al.^28^ Fleming et al.^29^ Tang et al.^32^ Paduano et al.^30^ Balinda et al.^31^	HSP90	**Pro‐tumoral** Antitumor immunity suppression via PMN‐MDSCs^28^, ^29^ Angiogenesis promotion via IKK/IκB/NF‐κB/CXCL1 axis in CAFs^32^ **Anti‐tumoral** C‐Raf pathway suppression via fhit protein interaction^30^ BRAF pathway inhibition via Hsp90/Cdc37‐MLK3 interaction^31^
Ke et al.^33^	HSP27	**Pro‐tumoral** Promoted cancer cell migration via Prion protein‐dependent depolymerization of F‐actin^33^
Cristofani et al.^34^ Yang et al.^35^	HSPB8	**Anti‐tumoral** Arrest of oncogenic cellular events and induction of autophagy in BRAF‐ and NRAS‐mutated melanoma cells^34^ Blockade of oncogenic PI3K/AKT/mTOR pathway via BAG3 interaction^35^
Peng et al.^42^ Muniz‐Bongers et al.^43^ Zaman et al.^52^	MMP2	**Pro‐tumoral** Antitumor immunity suppression via CAFs and via deleterious TLR2‐ and TLR4‐signaling^42^, ^43^ **Anti‐tumoral** Promotion of anti‐tumor T cell immunity by release of ECM structural protein mutants^52^
Tomonobu et al.^58^ Chen et al.^60^	S100A8/S100A9	**Pro‐tumoral** Upregulation of metastatic cellular events via S100A8/A9‐SSSRs'‐TLR4 axis^58^ Induction of pneumotropic metastasis via S100A8/A9‐MCAM axis^60^
Zeng et al.^62^	S100A11	**Pro‐tumoral** Transfer of metastatic competence via S100A11‐Sec23a and miR‐487a‐5p pathways^62^
Alasady et al.^69^ Wu et al.^67^ Roy Choudhury et al.^68^	S100B	**Pro‐tumoral** Anti‐tumor immunity suppression via IL‐6 signaling downregulation^69^ Blockade of p53 onco‐suppressive activity^67^, ^68^
Sun et al.^70^	hBD‐1 and hBD‐2	**Anti‐tumoral** hBD‐2 antiproliferative effects on human melanoma cells in vitro and hBD‐1 hypoexpression in melanoma patients^70^
Kiatsurayanon et al.^78^	Cathelicidin LL‐37	**Pro‐tumoral** Promotion of cellular oncogenic events via upregulation of EGFR and EGFR2 and tumor progression via YB‐1 and NF‐κB activation^78^ **Anti‐tumoral** Supporting the killing activity of NK cells^78^
Li Pomi et al.^79^	HMGB1	**Pro‐tumoral** Promotion of neoangiogenesis and pro‐tumoral immunity (via IL‐10‐producing M2 macrophages), upregulation of proinflammatory cytokines IL‐23 and IL‐17, and suppression of cytotoxic T‐cell activity (via the RAGE/HMGB1 axis)^79^
Wei et al.^81^	HMGN1	**Anti‐tumoral** Promotion of preferential Th1‐polarized antitumor immunity^81^
Yang et al.^41^ Wu et al.^87^ Okuyama et al.^88^ Gao et al.^90^ Schuijs et al.^91^ Dominguez et al.^92^ Lucarini et al.^93^ Andreone et al.^94^	IL‐33	**Pro‐tumoral** Promotes proliferation, migration and invasion of melanoma cells and VM tube formation through ST2^41^ Activation of macrophages which protect melanoma cells from killing mediated by TILs by upregulating the expression of MMP‐9 ^87,88^ **Anti‐tumoral** Activation of CD8^+^ T cell‐dependent mechanism^90^, ^91^, ^92^, ^93^, ^94^
Yao et al.^101^	TSLP	**Pro‐tumoral** Promoting melanoma progression and metastasis^101^
Benatar et al.^103^, ^104^	IL‐25	**Anti‐tumoral** Increase in serum levels of IL‐5 and increased numbers of eosinophils^103^, ^104^

Abbreviations: ATP, adenosine triphosphate; CAFs, cancer‐associated fibroblasts; CTL, cytotoxic T lymphocytes; CXCL1: C‐X‐C Motif Chemokine Ligand 1; ECM, extracellular matrix; EGFR, epidermal growth factor receptor; hBD, human β‐defensins; HMGB1, high mobility group box‐1; HMGN1, high mobility group nucleosome‐binding protein 1; HSP, heat shock protein; IKK, IκB kinase; IL, interleukin; MCAMs, melanoma cell adhesion molecules; MMP, matrix metalloprotease; NF‐κB, nuclear factor κB; NK, natural killer; PMN‐MDSCs, polymorphonuclear myeloid‐derived suppressor cells; RAGE, receptor for advanced glycation endproducts; ROCK, rho‐associated protein kinase; Th9, IL‐9‐producing CD4^+^ T‐helper; TIL, tumor‐infiltrating lymphocytes; TLR, toll‐like receptors; TSLP, thymic stromal lymphopoietin; VM, vascular mimicry.

## 3 CONCLUSIONS AND FUTURE DIRECTIONS

Based on the emerging literature discussed here, the alarmins molecular world undoubtedly represents a fascinating and pioneering field in which international scientific research should focus its efforts. This is especially true if alarmins are considered as a “mine” for elucidating new foundational pathogenetic mechanisms of cutaneous malignant melanoma and, more extensively, of other non‐melanoma skin cancers (NMSCs). From such a starting point, the boundless potential for clinical utility of the involved alarmins, with reference to diagnosis, differential diagnosis, prognosis, prediction of therapeutic response and, not least, improvement of the available therapeutics for counteracting the leading cause of and increased incidence of death for skin cancer, can be inferred. In this framework, the already mentioned strand of phytotherapeutic research, so far quite active only for MMPs and AMPs, are promising, along with the design of new alarmins‐targeting monoclonal antibodies and alarmins‐based anti‐melanoma vaccines, and the progress of pioneering miRNAs‐based drugs. To this end, the goals of the coming years should be oriented toward a more intensive research focus regarding the epigenetic mechanisms governing the expression of the debated alarmins in the TME, as well as on an increasingly conclusive elucidation of their pro‐ or anti‐tumor role. This would shed light on the still well‐represented dualisms and functional contradictions intrinsic to individual alarmins, not only in the context of melanoma, but also extended to the major NMSCs.

## FUNDING INFORMATION

None.

## CONFLICT OF INTEREST STATEMENT

None declared.
